# Responsive Behaviors and Pain Management in Hospital Dementia Care: A Before and After Comparison of the “Serial Trial Intervention”

**DOI:** 10.3389/fpain.2022.810804

**Published:** 2022-05-04

**Authors:** Albert Lukas, Melanie Bienas, Benjamin Mayer, Lukas Radbruch, Irmela Gnass

**Affiliations:** ^1^Competence Center of Geriatric Medicine, Helios Medical Center Bonn/Rhein-Sieg, Academic Teaching Hospital, University Bonn, Bonn, Germany; ^2^Department of Orthopedics and Trauma Surgery, University Hospital Bonn, Bonn, Germany; ^3^Institute of Epidemiology and Medical Biometry, University Ulm, Ulm, Germany; ^4^Helios Medical Center Bonn/Rhein-Sieg, Palliative Medicine, Academic Teaching Hospital, University Bonn, Bonn, Germany; ^5^Department of Palliative Medicine, University Hospital Bonn, Bonn, Germany; ^6^Paracelsus Medical University, Institute for Nursing Science and Practice, Salzburg, Austria

**Keywords:** pain, dementia, behavioral and psychological symptoms of dementia (BPSD), non-pharmacological and pharmacological intervention, acute care hospital, responsive behavior management

## Abstract

**Purpose:**

Responsive behavior, often referred to as behavioral and psychological symptoms of dementia (BPSD), is among the most critical disorders in dementia whereby nursing personnel in hospitals are increasingly confronted with such symptoms. The purpose was to reduce the level of BPSD in an acute hospital environment through a stepwise procedure followed by the initiation of a needs-oriented treatment.

**Methods:**

An open, prospective, interventional study with before-after comparisons was used to implement “Serial Trial Intervention” (STI) in three hospital wards (internal medicine, surgery, geriatric) after its adaption for hospital setting which was supplemented with a detailed pain assessment. Participants were 65 years and older. Potential causes of BPSD were clarified in a stepwise procedure and, if possible, eliminated. The primary outcome was the reduction in BPSD measured by the Neuropsychiatric Inventory (NPI-Q-12) while secondary outcomes were through the use of non-pharmacological and pharmacological interventions.

**Results:**

No significant reduction in NPI-Q-12 could be found. However, significantly more mobilizations and changes of position were carried out. Higher antipsychotic use was seen in the after-groups presumably due to the higher rates of delirium and cognitive impairment. Furthermore, the data showed no increase in analgesic use.

**Conclusion:**

No significant reduction in NPI-Q-12 was observed in the before-after study. The use of antipsychotics even increased most probably due to a higher incidence of deliriousness in the after-group. However, STI seemed to improve attention to underlying causes of BPSD as well as pain. Proof that STI leads to NPI-Q-12 reduction in hospitals is still pending.

## Key summary points

- **Aim** To reduce the level of behavioral and psychological symptoms of dementia (BPSD) in an acute hospital environment through a stepwise procedure following by the initiation of a needs-oriented treatment.
- **Findings** No significant reduction in Neurospychiatric Inventory (NPI-Q-12) could be found. However, significantly more mobilizations and changes of position were carried out. Higher antipsychotic use was seen in the after-implementation groups presumably due to the higher rates of delirium and cognitive impairment.
- **Message** Although final proof of the successful use of Serial Trial Intervention (STI) in the hospital setting is still pending, STI appears to be at least a tool for raising awareness regarding possible underlying causes of BPSD and pain as well as offering needs-based therapies.

## Introduction

Dementia-related diseases is one of the greatest challenges facing our aging population. They cause considerable stress for the affected person, caregivers and staff members, and lead to considerable economic burdens; for 2019, global costs have been estimated at $1 trillion (1). Up to 40% of people admitted to hospital live with dementia which often leads to a prolonged hospital stay (2). Responsive behavior in the course of in-patient treatment is frequently responsible for prolonging the stay. They lead to a considerable strain on affected patients and healthcare professionals, and disturb the daily scope of pratice in an acute care unit (3).

Responsive behavior, also known as challenging behavior as well as being a subset of BPSD (“Behavioral and Psychological Symptoms of Dementia) (4), is a heterogeneous group of symptoms and behaviors that can be broadly divided into affective symptoms, psychosis, hyperactivity, and euphoria (5). Examples of responsive behaviors include wandering, yelling, kicking and aggression. Up to 76% of people with dementia treated in hospitals suffer from BPSD (2). While the term “BPSD” reflects a more clinically diagnostic language, the term “responsive behavior” reflects a more person-centered language that emphasizes the dignity of the affected person. Furthermore, the term “challenging behavior” has a negative and misleading connotation and suggest that a problem is caused by an individual as an intrinsic deficit of a dementia (6–8).

The incidence of BPSD is influenced by patient-related factors such as the severity of cognitive impairment (9) as well as factors from a patient's environment and interaction with the caregiver (10). In this context, Algase et al. provide a possible explanation for responsive behavior in dementia in their needs-driven, dementia-compromised behavior (NDB) model (11). Thus, BPSD arises in the context of unsatisfied needs such as undetected pain (12–14). Affected people try to express their needs through words, movements or actions (6). Due to their inability to verbally express pain, pain recognition in people with advanced dementia is far more difficult, often leading to under-treatment of pain and increased BPSD (14). Consequently, it has been reported that optimization of analgesic treatment may lead to a reduction in BPSD (15).

Considering the increasing number of affected patients with BPSD in hospital settings, the task is to improve the diagnosis and therapy of responsive behaviors. One possibility to significantly improve the situation is seen in a structured clarification of the causes of BPSD followed by a specific therapy tailored to meet the needs. For this purpose, Kovach et al. (16) developed the so-called Serial Trial Intervention (STI), a structured step-by-step clarification process for apparently unexplainable behavior in people with dementia (PWD). According to the trial-and-error principle, possible reasons for responsive behavior are clarified and, if possible, eliminated through appropriate interventions. While, in the meantime, STI has been successfully used in nursing homes (17), a recent review revealed that none of the 24 projects studied had yet been implemented in an acute care hospital setting (18). To enable its use in an acute care hospital setting, an adapted version of STI for a hospital setting was developed in the present study with particular attention paid to pain as an important potential cause of responsive behavior which was subsequently applied in an acute care hospital. The effect on STI was measured through changes in BPSD using the Neuropsychiatric Inventory Questionnaire (NPI-Q-12) (19, 20), a validated instrument covering 12 neuropsychiatric domains. As not each of the 12 items are equally influenced by the STI, subscales for agitation/aggression, mood and frontal behavior were additionally used (21).

Hence, the aim of the present study was to test the following hypothesis:

A structured stepwise clarification of possible causes for apparently unexplainable behavior in people with dementia (PWD), as represented by the STI, also leads to a more cause-related therapy in an acute hospital environment, such as increased pain therapy in cases of pain and lower use of antipsychotics.

The following questions should be answered in greater detail.

1) In terms of effectiveness, does the use of the adopted version of STI lead to a significant reduction in responsive behavior in the after-implementation group compared to the before-implementation group within a four-day observation period, measured as a score reduction of the Neuropsychiatric Inventory Questionnaire (NPI-Q-12)? (Primary outcome)
2) Are there any significant reductions in the NPI-Q subscales “Agitation/Aggression,” “Mood,” or “Frontal” using the adopted version of STI under the assumption that not all NPI-Q items are equally affected by STI?
3) Can the increased use of non-pharmacological interventions to treat responsive behavior be observed through the use of STI?
4) Is there an increased use of analgesics in the sense of a more cause-related treatment of responsive behavior in the case of underlying pain through the application of STI within the four-day observation period?
5) Can the administration of non-specific psychotropic drugs be reduced at the same time and what is the situation regarding sedatives and antidepressants?
6) Generally speaking, is an adapted version of STI basically feasible and applicable in a hospital setting?

## Methods

### Study Design

An open, prospective, interventional study in form of an inter-period comparative design with a before-after comparison was conducted in a general hospital in Germany. One study group was recruited before and another after introduction of a stepwise cause assessment for BPSD. The before-implementation phase was designed to gain knowledge about the incidence and severity of BPSD, especially concerning responsive behavior on three different wards (internal medicine, surgery, and geriatric) while the after-implementation phase was used to evaluate the effect of a structured clarification of causes and initiation of a specific treatment.

### Intervention

The intervention consisted of the introduction of the Serial Trial Intervention (STI) with special attention being paid to pain. STI works according to the trial-and-error principle. After recognizing BPSD, the possible cause is investigated in a structured step-by-step process (five steps: physical assessment, affective assessment, non-drug treatment, analgesic and antipsychotic treatment). If a supposed cause is found, an attempt is made to eliminate it (e.g., emptying the full bladder). If there is no significant improvement (50% reduction of the BPSD is required), the next step is taken. In the final step, training also included convening a case conference for the decision to administer antipsychotics. The intervention checklist was created and developed in the context of an anonymous survey of nurses from three different wards (internal, geriatric, surgery) by means of a semi-structured interview with open and closed questions about BPSD and possible therapeutic countermeasures as part of a kick-off meeting on dealing with BPSD and especially concerning responsive behavior (3). The list was supplemented by further training materials provided by the nursing experts involved which was recently published (22). The observation period of each PWD in the before and after-phases, respectively, was set at 4 days. Usually, it takes some time until a stable improvement in BPSD can be achieved. In addition, STI is characterized by a stepwise approach. It is also not consistently expected that the first intervention chosen will definitely lead to an improvement in BPSD. In this respect, we chose a follow-up period during whereby STI was applied daily. This daily application served, on the one hand, to control whether the applied intervention showed the effect in BPSD and, on the other, to determine the necessity for a necessary modification of the therapy. Thus, the 4-day observation period was guided by the consideration of providing sufficient time for STI to show possible effects, but also by clinically practical considerations, based on the experience that, within this time, the majority of those affected should show signs of improvement in their BPSD. To highlight a possible difference in terms of BPSD in the before-after comparison, the first and fourth days of the intervention were defined as the main measurement time points of the analysis.

### STI-Training

Scientific experts in the field of STI and pain (I.G, J.N., C.S, and A.L.) supported the development of a curriculum. Between the before- and after-implementation phase, a specific trainee programme based on STI and including pain assessment, conducted by a registered nurse and a physician who are both qualified in gerontology, took place. As basic training, all healthcare professionals (registered nurses, physicians, and therapists) on the three wards received a 90-min training course. In addition, so-called 1-day mentor training sessions (8 h) were held for individual employees as support for the briefly trained employees. The training content included, in particular, the recognition, importance and background of BPSD, STI as a clarification process with its five steps (physical assessment, affective assessment, non-drug treatment, tentative administration of an analgesic, and tentative administration of psychotropic drugs), the influence of pain in BPSD, intensive STI application training and pain assessment for PWD as well as a checklist of potential non-pharmacological and pharmacological treatments.

### STI Adoption to Accommodate the Hospital Setting

Originally developed as a systematic process to proactively reduce BPSD in nursing homes (16, 17)—whereby the original text was translated into German (23)—STI first had to be adapted to suit the specific requirements of the hospital setting. For example, it can be assumed that in contrast to the original version for a nursing home environment, more injuries, post-operative conditions or overall conditions associated with pain can be expected in a clinic. To make this clear, the nurses were made aware of this fact during the training. Likewise, attention was paid to whether the behavior occurred during specific activities (e.g., nursing procedures).

### STI Supplements “Structured Pain Assessment”

Particular emphasis was placed on pain assessment. The original STI only hints at considering pain as a possible cause for an unexplainable behavior in addition to physical and affective disorders and to initiate pain therapy if necessary. Details, such as how pain should be measured, especially in light of the fact that many people on whom the STI is to be used are cognitively impaired, are missing. STI was initially supplemented by the Verbal Rating Scale (VRS) as a self-assessment instrument for pain intensity—for people with mild to moderate cognitive impairment—and, secondly, by a proxy-assessment for pain—the PAINAD scale—for people with advanced dementia (24).

### Primary Outcome

The primary outcome was defined as the reduction in BPSD, as measured using the Neuropsychiatric Inventory Questionnaire (NPI-Q-12), in the after-implementation group compared to the before-implementation group within the observation period of 4 days.

### Secondary Outcomes

Secondary outcomes were defined as a reduction in BPSD, measured using NPI-Q subscales, by the use of non-pharmacological treatments as well as consumption of analgesics, antipsychotics, sedatives and antidepressants before and after the introduction of STI, and the feasibility of STI in a hospital setting.

### Inclusion and Exclusion Criteria

Suitable patients were 65 years and older who had a Mini Mental State Examination (MMSE) ≤ 24, assigned a minimum assumed 4-day length of stay (a decision based on the principal diagnosis which can be assigned to a specific mean length of stay in Germany) at one of the three intervention wards and who showed signs of BPSD (at least one of the 12 possible symptoms according to NPI-Q). All suitable patients received written information and were asked for participation through informed consent. Where informed consent was not possible, this was provided by their legal guardian or authorized person.

### Measurements

#### Neuropsychiatric Inventory Questionnaire

BPSD was measured using the Neuropsychiatric Inventory Questionnaire (NPI-Q-12), a validated instrument for evaluating psychopathology in dementia. The NPI-Q-12 is a brief clinical form of the Neuropsychiatric Inventory (NPI) (19, 20). The questionnaire covers 12 neuropsychiatric areas (Delusion, Hallucination, Agitation/Aggression, Depression/Dysphoria, Anxiety, Elation/Euphoria, Apathy/Indifference, Disinhibition, Irritability/Lability, Motor Disturbance, Night-time Behavior, and Appetite/Eating). The NPI-Q-12 has good test-retest reliability and convergent validity, correlating with the full NPI at 0.9 (20). Due to the inability of many participants to use the NPI-Q as a self-administered questionnaire, we used the instrument with a proxy rating. Each of the 12 areas was assessed by using a screening question. The severity of the symptoms was determined using a three-level Likert scale (1-mild, 2-moderate, 3-severe). The total NPI-Q-12 severity score represents the sum of individual symptom scores ranging from 0 to 36. In order to detect significant changes regarding BPSD, the NPI-Q-12 was applied once a day over the four observation days. The NPI-Q-12 was determined by the study nurse together with the responsible nurses on the wards.

In recent years, there have been recurrent efforts to identify clusters or syndromes of NPI that represent significant subgroups of patients with different neuropsychiatric syndrome constellations. The goal of these efforts has been to give researchers and clinicians more options for individualized care of specific NPI clusters and to better control their therapeutic interventions. Thus, Trzepacz et al. (21) developed and validated three different NPI-Q subscales for agitation/aggression, mood, and frontal syndromes. The compositions were determined based on publications on descriptive population data, and from exploratory factor analyses as well as known phenotypic and neuroanatomical relationships among symptoms drawn from larger neuropsychiatric publications (21). The subscales can be generated from the NPI-Q-12, with the NPI-Q-4-Agitation/Aggression subscale comprised of four items: Agitation/Aggression, Disinhibition, Irritability/Lability, and Motor Disturbance. The NPI-Q-3-Mood subscale consists of three items: Depression/Dysphoria, Anxiety, and Irritability/Lability while the NPI-Q-4-Frontal Subscale comprises Elation/Euphoria, Apathy/Indifference, Disinhibition, and Irritability/Lability. It is conceivable that not all items of the NPI-Q-12 are equally influenced by STI. For example, the NPI-Q-12 includes behavior such as appetite/eating and elation/euphoria that STI would presumably not impact. Thus, in an extended analysis, NPI-Q subscales would also be considered.

#### Pain Assessment

The original STI was supplemented by a stepwise pain assessment with a self-assessment tool (VRS) and proxy-assessment tool (PAINAD). The VRS, a four-level Likert scale (none, mild, moderate, severe pain), is one of the preferred self-report pain assessments for detecting pain in older people and is easy to use with reliable measurements even more so in people with moderate cognitive impairment (25). The PAINAD scale is also one of the most frequently recommended pain assessment scales in advanced dementia, clinically useful, easy to perform and with sufficiently good psychometric values (26). This proxy-assessment instrument consists of five observable items (breathing, negative vocalization, facial expression, body language, and reaction to consolation), where each item can be rated between 0 and 2 thus resulting in a maximum score of 10 points. The pain cut-off level in PAINAD is a controversial issue in publications. Zwakhalen et al. suggested a value of ≥2 (27). Other authors were able to determine a value of ≥4 as the cut-off for pain in a comparison with a self-report pain assessment (28). In the present work, we opted for a more conservative estimate (cut off ≥ 4), because we assumed a greater probability of relevant pain being present at a higher value. Both pain assessments were integrated parts of STI and applied by the respective ward nurses once daily over the four observation days. The selection of the appropriate pain assessment was decided by a nurse according to the patient's cognitive abilities at the time of assessment. In accordance with the general recommendations of pain measurement, a self-assessment was always initially attempted. Only in those cases where this was not reliably possible was the PAINAD scale used. Pain was measured both during rest and movement. In each case, the highest pain intensity measured over the four-day observation period was included in the analysis. This was usually a pain measurement during movement whereby transfer situations were mostly used for this purpose (26).

Pain was also measured in the “before” group but was not as structured as with the modified STI in the “after” group. In addition to VRS, the Numerical Rating Scale (NRS) was used in this group. NRS is a pain scale with numerical values from 0 (= no pain) to 10 (= maximum imaginable pain). It is known from publications that NRS is less suitable for cognitively impaired older people in relation to VRS (25). However, in the “before” group we did not want to influence the choice of pain assessment.

#### Treatment Documentation

Non-pharmacological treatment of BPSD was recorded daily within the 4 days of observation. A checklist including different procedures such as pacifying talk, providing assistance with mobilization, offering food/drinks or other activities like listening to music, was used.

Permanent pharmacological treatment was documented on each of the four observation days, focusing on tranquilizers, antidepressants, psychotropics, and analgesics. Analgesics were classified according to their WHO level and psychotropic drugs according to their neuroleptic potency.

Nurses applied non-pharmacologic treatments or initiated pharmacologic interventions through consultation with physicians. The treatments were documented by the study nurses who were in close contact with the nurses on site. According to STI steps, priority was given to non-pharmacological interventions.

#### Assessments to Describe the Study Population

A wealth of assessments served to describe the study population in more detail and were collected by a member of the research team (study nurse).

Cognition was assessed by means of MMSE (29). It measures cognitive function especially in terms of orientation and memory. The score ranged from 0 to 30. MMSE scores < 24 indicate the presence of dementia (30).

Functional Assessment Staging (FAST) reflects functional deterioration throughout the course of dementia (31, 32). FAST is an interview-based global severity scale for clinicians, measuring progression of dementia on the basis of cognition-based functioning (33). It is comprised of seven major functional levels (from I to VII) and an additional 11 sub-stages corresponding to Stage VI (a-e = moderately severe dementia) and VII (a-f = severe dementia). The higher the score, the more severe functional deterioration and cognitive impairment is seen. MMSE and FAST were determined exclusively on the first day.

The Delirium Observation Scale (DOS) is a valid instrument used to detect delirium with high sensitivity (90%) and specificity (91%) (34, 35). For this study, the shortened 13-item version was used. Item scores equal to or higher than 3 points (out of 13) indicate a state of delirium. The DOS was assessed on the first and on the fourth observation day. Changes during the observation period were calculated using these two measurement points.

The four-item Geriatric Depression Scale (GDS-4) is suitable as a screening tool for detecting the risk of depression in older people. It is easy to administer and has a high degree of sensitivity and specificity. A score equal to or higher than 2 indicates a risk of depression (36, 37).

Patient functionality was assessed by using Barthel's Index (ADL). This 10-point scale measures a patient's degree of autonomy in daily living activities with a total score ranging from 0 to 100. Lower scores indicate higher levels of dependency (38). GDS as well as Barthel's Index were determined exclusively on the first day.

### Ethical Considerations

Ethical approval for the study was obtained by the Ethics Committee, University Bonn, Germany (No. 252/15). An ongoing informed consensus enabled the participants to communicate their non-participation at any time.

### Statistical Analysis

The analysis was designed as a before-after comparison. Regarding the description of the study population, absolute and relative frequencies have been given. Differences between the before and after-implementation group were tested using a chi-squared test or the Fisher exact test for categorical variables and for normally distributed continuous variables with the *t*-test for independent samples or the Mann-Whitney *U*-test in case for skewed distributions. The analysis included the first and fourth observation days of the patients from the two study groups. For the primary outcome, evaluation was designed to take into account the specificity of STI with its wide range of possible intervention methods, and the fact that STI is individually tailored to meet the needs of those affected which inevitably leads to heterogeneity in the measured “intervention effect.” Thus, in addition to a restricted look at NPI-Q-12 on specific days by determining median NPI-Q-12 values for treatment Days 1 and 4, the “individual treatment effect” by means of a difference in NPI-Q-12 (d1-d4 difference) in order to form categories from improved to deteriorated was determined. Median NPI-Q-12 values together with their range are reported for each assessment day. In addition, “changes in the NPI-Q-12 score between Day 1 and 4,” e.g., due to a pharmacological intervention, were categorized (improvement, deterioration, unchanged findings) per patient in each examination group. For the comparison of both cohorts, a chi-square test was applied to this categorized assessment of NPI-Q-12 change from Day 1 to 4. All analyses were performed identically for both the NPI-Q-12 version and its three subscales: NPI-Q-4-Agitation/Aggression, NPI-Q-3-Mood and NPI-Q-4-Frontal.

Furthermore, in order to address the possibility of a confounded result, linear mixed model analysis was used to assess the potential impact of unequally distributed variables in the before and after cohorts on NPI-Q-12 scores using repeated measures data assessed on Days 1 and 4. In general, a *p*-value < 0.05 was considered to be statistically significant whereas the study design did not allow for a confirmatory interpretation of the findings.

All analyses were performed using IBM SPSS^®^ Statistics for Windows version 26.0, Armonk, New York, USA. Microsoft Excel^®^ 2019 was used for the creation of graphs.

## Results

### Study Sample

**Figure 1:**
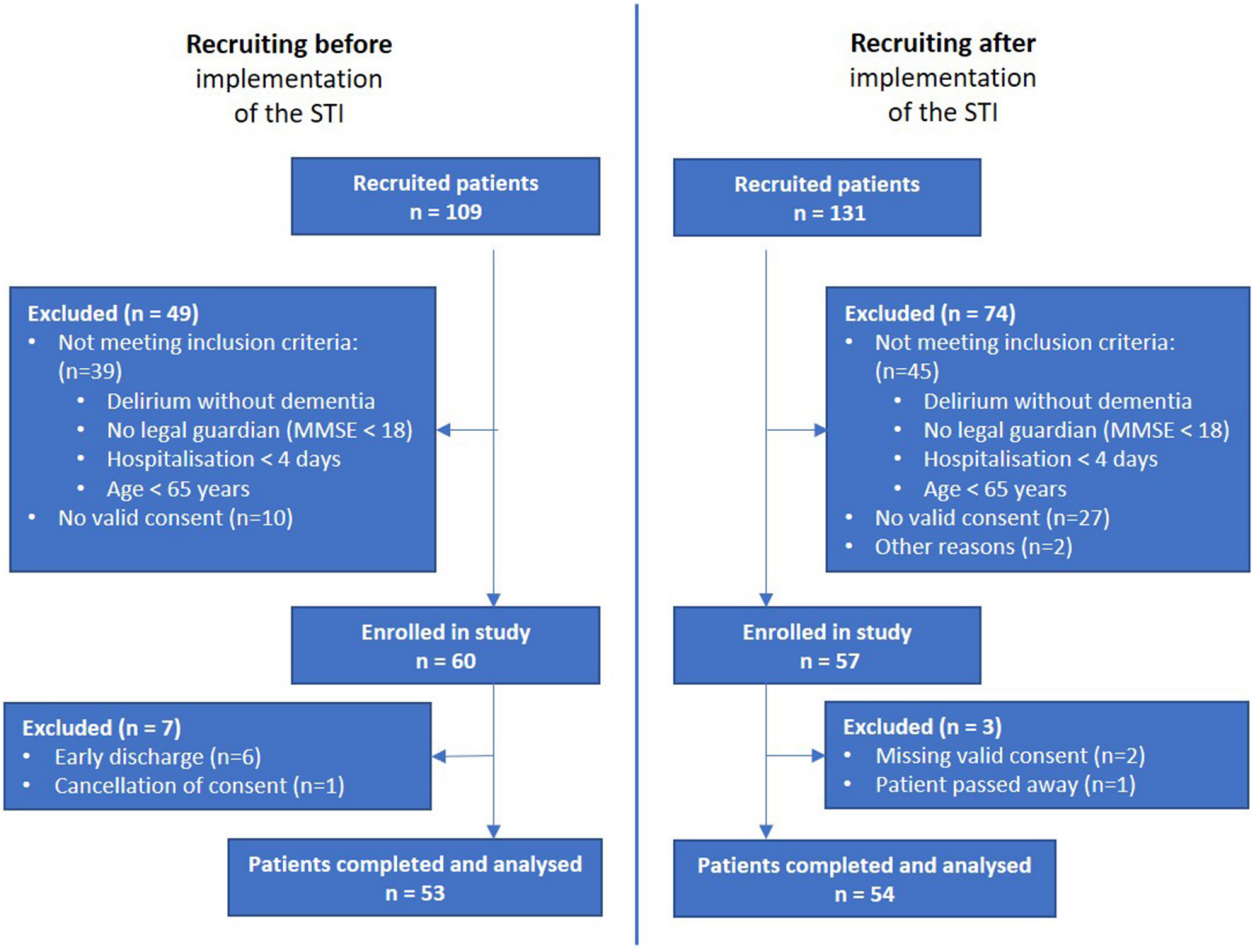
Study population.

From the initial 109 patients recruited from the before-implementation group, those not meeting inclusion criteria (*n* = 39), with no consent given (*n* = 10), with early discharge (*n* = 6) and cancellation of consent (*n* = 1) were excluded, leading to a before-interventional study population of 53 participants. From the initial 131 patients taken from the after-implementation group, those not meeting the inclusion criteria (*n* = 74), with no valid consent (*n* = 27), other reasons (*n* = 2), missing valid consent (*n* = 2) and deceased patient (*n* = 1) were excluded, leading to an after-interventional study population of 54 participants. In total, the recruitment process took 21 months. Recruitment for the “before” group was set at 6 months in advance. Training sessions then took place, followed by the introduction of the STI. In the “after” group, the recruitment process for a comparable number of patients took a significantly longer 15 months. The final analysis included 107 participants, 53 in the before-implementation group and 54 in the after-implementation group (Figure 1).

### Sample Characteristics

No significant difference between the two study groups was found regarding age, gender, distribution of the participants in the three wards, ADL functions and MMSE (Table 1). A relevant difference could however be seen in the Functional Assessment Staging (FAST) with considerably higher functional deterioration in the after-group [VIb (II-VIIa) vs. Vie (Via-VIId), *p* =.009]. The after-implementation group showed significantly more frequent indications of delirium [19 (35.8%) vs. 47 (87.0%), *p* < 0.001) and had a higher proportion of participants who did not experience any change in their delirium among the interventions initiated. There was no significant difference regarding the type of dementia, signs of depression, pain, maximum pain intensity, analgesic therapy, and duration of hospital stay.

**Table 1:** Characteristics of the study population before and after STI implementation.

	**Before implementation *n* = 53 (%)**	**After implementation *n* = 54 (%)**	***P*-value**
Age, years (mean ± SD)	84.72 ± 5.49	84.09 ± 6.58	.60 *T*-test
Female gender	37 (69.8%)	32 (59.3%)	0.25
Ward			
Geriatric	24 (45.3%)	27 (50.0 %)	0.53
Internal medicine	19 (35.8%)	21 (38.9%)	
Surgery	10 (18.8%	6 (11.1%)	
ADL^a^			0.73
Assistance required	16 (30.2%)	18 (33.3%)	
Dependent	37 (69.8%)	36 (66.7%)	
GDS-4^b^			0.91
Depression	10 (18.9%)	12 (22.2%)	
No depression	18 (34.0%)	23 (42.6%)	
DOS^c^	19 (35.8%)	47 (87.0%)	**<0.001**
Change^d^ within 4 days			**0.02**
No change	6 (11.3%)	29 (53.7%)	
Improvement	12 (22.6%)	12 (22.2%)	
Deterioration	1 (1.9%)	6 (11.1%)	
Type of dementia			
Alzheimer's	5 (9.4%)	8 (14.8%)	0.39
Vascular dementia	4 (7.5%)	6 (11.1%)	0.74
Unknown	43 (81.1%)	40 (74.1%)	0.38
MMSE^e^ (median, min-max)	16 (5–24)	13.5 (0-21)	0.08 *U*-test
Mild	14 (26.4%)	8(14.8%)	0.08
Moderate	11 (20.8%)	22 (40.7%)	
Severe	6 (11.3%)	6 (11.1%)	
FAST^f^ (median, min-max)	VIb (II-VIIa)	VIe (Via-VIId)	**0.009** *U*-test
Pain^g^	43 (81.1%)	39 (72.2%)	0.28
Maximum pain intensity^h^ (median, min-max)	3 (0-8)	3 (0-9)	0.32 *U*-test
Analgesic therapy^i^	38 (88.4%)	33 (84.6%)	0.62
Duration of hospital stay (median, min-max)	21 (6–54)	18 (5–54)	0.50 *U*-test

a *Activities of daily living: assistance required is defined by ADL hierarchical scale score 35-80, dependent on an ADL hierarchical scale score 0-30*.

b *Geriatric Depression Scale, evaluable data. Depression is assumed by GDS ≥ 1*.

c *Delirium Observation Scale. Delirium is assumed by DOS > 3*.

d *Change of DOS-Score over a period of 4 days. Measurement points were the first and fourth observation days*.

e *Mini Mental State Examination, evaluable data. Mild is defined by MMSE 18-24, moderate by MMSE 10-17, severe by MMSE < 10*.

f *Functional Assessment Staging*.

g *Pain: Minimum of mild pain in pain assessment or current pain therapy in the respective overall study groups. NRS values converted to VRS equivalents*.

h *Maximum pain intensity over a period of 4 days among patients suffering from pain (pre: n = 43/post: n = 39)*.

i *Prescribed analgesics among patients suffering from pain (pre: n = 43/post: n = 39). Significant results (p < 0.05) are highlighted. Unless otherwise stated, the chi-squared test was performed. Significant values are in bold*.

### Neuropsychiatric Symptoms

Regarding the primary outcome—a reduction in BPSD in the after-implementation group compared to the before-implementation group within the observation period of 4 days in each case—no significant reduction was found (Table 2). Neither the direct comparison of the two study groups using the categorized change assessment between Days 1 and 4, nor the findings based on a linear mixed model adjusted for FAST and DOS values indicated a significant effect of STI on the NPI-Q. Although DOS, in contrast to FAST, showed a statistically significant effect on the NPI-Q and must therefore be treated as an important confounder, its plain effect was negligible (only half of a point on the NPI-Q-12 scale). Based on the results of the linear mixed model analysis there is an additional non-significant tendency of slightly decreased NPI-Q-12 values in the post-cohort when compared to the pre-cohort, but the plain effect was also only about half of a point on the NPI-Q-12 scale. Summing up, the results confirm that no meaningful effect of STI on the NPI-Q-12 was detectable. In the extended subgroup analysis (NPI-Q-4-Agitation/Aggression, NPI-Q-3-Mood, and NPI-Q-4-Frontal) no significant differences were found between the study groups as well.

**Table 2:** Severity of NPI-Q symptoms and changes within the study population on Days 1 and 4 before and after STI implementation, for NPI-Q-12 and NPI-subscores.

**NPI-Q-12 score^a^**	**Before implementation *n* = 53 (%)**	**After implementation *n* = 54 (%)**	***P*-value**
**a) Unadjusted analysis**			
Day 1 (median, min-max)	9 (1–25)	9 (2–25)	0.90
Day 4 (median, min-max)	5 (0-29)	7 (0-22)	0.08
Change^b^ within 4 days:			0.34 Fisher test
No change	2 (3.8%)	4 (7.4%)	
Improvement	38 (71.7%)	31 (57.4%)	
Deterioration	13 (24.5%)	19 (35.2%)	
**NPI-Q subscales:**			
**NPI-Q-4-agitation/aggression score^c^**			
Day 1 (median, min-max)	5 (0-12)	5 (0-12)	0.65
Day 4 (median, min-max)	3 (0-12)	3 (0-12)	0.07
Change^b^ within 4 days:			0.10 χ^2^ test
No change	5 (9.4%)	11 (7.4%)	
Improvement	37 (69.8%)	27 (50.0%)	
Deterioration	11 (20.8%)	16 (29.6%)	
**NPI-Q-3-Mood score^d^**			
Day 1 (median, min-max)	2 (0-8)	2 (0-8)	0.90
Day 4 (median, min-max)	2 (0-9)	2 (0-8)	0.48
Change^b^ within 4 days:			0.12 χ^2^ test
No change	16 (30.2%)	8 (14.8%)	
Improvement	24 (45.3%)	26 (48.1%)	
Deterioration	13 (24.5%)	20 (37.0%)	
**NPI-Q-4-Frontal score^e^**			
Day 1 (median, min-max)	2 (0-9)	2 (0-9)	0.23
Day 4 (median, min-max)	2 (0-7)	2 (0-9)	0.42
Change^b^ within 4 days:			0.26 χ^2^ test
No change	14 (26.4%)	19 (35.2%)	
Improvement	25 (47.2%)	17 (31.5%)	
Deterioration	14 (26.4%)	18 (33.3%)	
	**Estimate**	**Standard error**	***P*** **-value**
**b) Adjusted analysis^f^**			
Day (1 vs. 4)	1.79	0.65	0.007
Group (before vs. after)	0.57	0.97	0.562
DOS	0.60	0.17	0.001
FAST	−0.06	0.19	0.740

a *Each symptom was graded according to the severity using the following scale: mild = score 1, moderate = 2 and severe = 3. Scores range from minimum 0 to maximum 36*.

b *Change of NPI-Q-Score over a period of 4 days. Measurement points were the first and fourth observation days (NPI-Q-Scores d1-d4 and subsequent categorization into improvement, deterioration, unchanged findings)*.

c *Sub-score for “Agitation and Aggression,” consisting of the four NPI items: Agitation/Aggression, Disinhibition, Irritability/Lability, Motor Disturbance*.

d *Subscore for “Mood,” consisting of the three NPI items: Elation/Euphoria, Apathy/Indifference, Irritability/Lability*.

e *Subscore for “Frontal,” consisting of the four NPI items: Elation/Euphoria, Apathy/Indifference, Disinhibition, Irritability/Lability*.

f *Adjusted analysis based on a linear mixed model including the NPI-Q-Score as the dependent and cohort (before vs. after) as well as DOS (Delirium Observation Scale) and FAST (Functional Assessment Staging) as predictor and confounding variables respectively. Unless otherwise stated, the U-test was performed in the unadjusted analysis*.

The incidence of the twelve NPI-Q symptoms vary widely. Agitation/aggression (in both study groups ~90%), night-time behavior disturbances (~80%), irritability (~75%) and anxiety (~57%, ~78%, respectively) were the most common. Anxiety and aberrant motor behaviors were significantly more common in the after-implementation group [30 (56.6%) vs. 42 (77.8%), *p* = 0.02, 35 (66.0%) vs. 45 (83.3%), *p* = 0.04, respectively] while apathy was observed more often in the before-implementation group [34 (64.2%) vs. 24 (44.4%), *p* = 0.04]. All other symptoms (delusion, hallucination, agitation/aggression, depression/dysphoria, euphoria, disinhibition, irritability, night-time behavior disturbances as well as appetite and eating abnormalities) occurred with equal frequency in the two study groups.

### Non-pharmacological Treatment

With regard to non-pharmacological measures, the most frequent was a calming conversation with the PWD. Mobilization was used significantly more often in the after-implementation group [23 (43.4%) vs. 44 (81.5%), *p* < 0.001] similar to change of position in bedridden PWD [21 (39.6%) vs. 38 (70.4%), *p* = 0.001] and offering drinks or food to overcome needs-driven behavior [8 (15.1%) vs. 24 (44.4%), *p* = 0.001] (Table 3).

**Table 3:** Non-pharmacological interventions before and after STI implementation.

**Non-pharmacological interventions**	**Before implementation *n* = 53 (%)**	**After implementation *n* = 54 (%)**	***P*-value**
Calming talk	52 (98.1%)	54 (100%)	0.50
Mobilization	23 (43.4%)	44 (81.5%)	**<0.001**
Change of positioning	21 (39.6%)	38 (70.4%)	**0.001**
Offering food / drinks	8 (15.1%)	24 (44.4%)	**0.001**
Basal stimulation	0 (0.0%)	0 (0.0%)	n.a.^a^
Interaction in groups^b^	9 (17.0%)	15 (27.8%)	0.18
Application of warmth / cold	0 (0.0%)	1 (1.9%)	1.0 Fisher
Massage	0 (0.0%)	0 (0.0%)	n.a.
Involvement/notice to ward physician	14 (26.4%)	20 (37.0%)	0.24
Involvement of relatives	7 (13.2%)	6 (11.1%)	0.74
Activities^c^	3 (5.7%)	4 (7.4%)	1.0 Fisher

a *Not applicable*.

b *“living room” (only on the geriatric ward)*.

c *e.g., handwork, reading. Significant results (p < 0.05) are highlighted. Unless otherwise stated, the chi-squared test was performed. Significant values are in bold*.

### Pharmacological Treatment

The presumed additional use of analgesics with STI including pain assessment tools did not occur. There were no significant differences in the before-after comparison of the analgesics used on the first or the fourth observation day (Table 4). Similarly, the before-after comparison of antipsychotics or sedatives on the first and fourth observation days showed no significant difference. There was no lower usage of antipsychotics on the fourth observation day as had been expected. Antidepressants were used significantly more often in the before-implementation group both on the first and fourth day of the study [19 (35.8%) vs. 8 (14.8%), *p* = 0.02 vs. 20 (37.7%) vs. 9 (16.7%), *p* = 0.02].

**Table 4:** Pharmacological intervention of study participants before and after STI implementation, Day 1 and Day 4.

	**Medication Day 1**			**Medication Day 4**		
	**Before implementation *n* = 53 (%)**	**After implementation *n* = 54 (%)**	***P*-value**	**Before implementation *n* = 53 (%)**	**After implementation *n* = 54 (%)**	***P*-value**
Analgetics^a^			0.81			0.36
None	14 (26.4%)	19 (35.2%)		13 (24.5%)	20 (37.0%)	
WHO^b^ I	10 (18.9%)	9 (16.7%)		10 (18.9%)	5 (9.3%)	
WHO II or III	5 (9.4%)	4 (7.4%)		5 (9.4%)	4 (7.4%)	
WHO I and II or III	24 (45.3%)	22 (40.7)		25 (47.2%)	25 (46.3%)	
Antipsychotics^c^			0.84			0.10
None	24 (45.3%)	24 (44.4%)		22 (41.5%)	23 (42.6%)	
Low potency	4 (7.5%)	6 (11.1%)		5 (9.4%)	5 (9.3%)	
Middle potency	11 (20.8%)	8 (14.3%)		11 (20.8%)	5 (9.3%)	
High potency	6 (11.3%)	5 (9.3%)		6 (11.3%)	2 (3.7%)	
Combination^d^	8 (15.1%)	11 (20.4%)		9 (17.0%)	19 (35.2%)	
Sedatives^e^	2 (3.8%)	5 (9.3%)	0.44	3 (5.7%)	5 (9.3%)	0.72
Antidepressants^f^	19 (35.8%)	8 (14.8%)	**0.02** **χ**^2^ **test**	20 (37.7%)	9 (16.7%)	**0.02** **χ**^2^ **test**

a *WHO I (metamizole, paracetamol, ibuprofen), WHO II (tramadol, tilidine/naloxone), WHO III (morphine, buprenorphine, fentanyl, hydromorphone, oxycodone)*.

b *World Health Organization analgesic ladder*.

c *Typical and atypical antipsychotics (prothipendyl, melperone, pipamperone, quetiapine, risperidone, clozapine, olanzapine, haloperidol)*.

d *Combination of low and middle or low and high potency antipsychotics*.

e *Sedatives (Lorazepam, oxazepam, zopiclone)*.

f *Antidepressant (duloxetine, mirtazapine, citalopram, venlafaxine, sertraline). Significant results (p < 0.05) are highlighted. Unless otherwise stated, the Fisher test was performed. Significant values are in bold*.

Changes in pharmacological intervention (increase and decrease) during the observation period are listed in Table 5. The summary of pharmacological intervention over the 4 days of observation showed no significant differences regarding analgesics and sedatives. Antidepressants did show a significant *p*-value [19 (35.8%) vs. 8 (14.8%), *p* = 0.02] but this can be primarily explained by the differences in antidepressant prescribing that already existed on the first day of the study in both study groups. This was different for antipsychotics. Although the comparison of antipsychotics on the first as well as on the fourth day showed no difference in before-after comparisons, the summary of pharmacological interventions showed a significant increase in antipsychotic drug use in the after-implementation group during the observation period [2 (3.8%) vs. 12 (22.2%), *p* = 0.02].

**Table 5:** Changes in pharmacological intervention (increases and decreases) over the four observation days, before and after STI implementation.

**Medication**	**Before implementation *n* = 53 (%)**	**After implementation *n* = 54 (%)**	***P*-value**
Analgetics^a^			0.29
Not applicable^b^	11 (20.8%)	15 (27.8%)	
Unchanged	37 (69.8%)	31 (57.4%)	
Increased^c^	4 (7.5%)	8 (14.8%)	
Decreased^d^	1 (1.9%)	0 (0.0%)	
Antipsychotics^e^			**0.02**
Not applicable	22 (41.5%)	22 (40.7%)	
Unchanged	27 (50.9%)	19 (35.2%)	
Increased^f^	2 (3.8%)	12 (22.2%)	
Decreased^g^	2 (3.8%)	1 (1.9%)	
Sedatives^h^			0.44
Not applicable	50 (94.3%)	49 (90.7%)	
Unchanged	2 (3.8%)	5 (9.3%)	
Increased	1 (1.9%)	0 (0.0%)	
Antidepressants^i^			**0.02**
Not applicable	33 (62.3%)	45 (83.3%)	
Unchanged	19 (35.8%)	8 (14.8%)	
Increased	1 (1.9%)	1 (1.9%)	

a *WHO I (metamizole, paracetamol, ibuprofen), WHO II (tramadol, tilidine/naloxone), WHO III (morphine, buprenorphine, fentanyl, hydromorphone, oxycodone)*.

b *Patients without the specific drug*.

c *Increase in analgesics over the 4 observation days*.

d *Decrease in analgesics over the 4 observation days*.

e *Typical and atypical antipsychotics (melperone, pipamperone, quetiapine, risperidone, clozapine, olanzapine, haloperidol)*.

f *Increase in antipsychotics over the 4 observation days*.

g *Decrease in antipsychotics over the 4 observation days*.

h *Sedatives (Lorazepam, zopiclone)*.

i *Antidepressants (Duloxetine, mirtazapine, citalopram, venlafaxine, sertraline). Significant results (p < 0.05) are highlighted. The Fisher test was performed. Significant values are in bold*.

## Discussion

### Neuropsychiatric Inventory

The basic idea of the project was that an apparently inexplicable behavior in a person with dementia very often has an identifiable reason. Evaluation of potential causes and needs-oriented treatment along the Serial Trial Intervention (STI) may help to eliminate these causes and reduce responsive behavior of people with dementia in an acute care hospital.

We expected a reduction in BPSD, measured as NPI-Q-12 as the primary outcome. However, no significant improvement in neuropsychiatric symptoms was found in the after vs. before-implementation group during the four-day observation periods, neither by using the NPI-Q-12 version nor by using NPI-Q subscales for agitation/aggression, mood or frontal symptoms. The reason for this observation could be the fact that both study groups were not as equivalent as initially expected. In this context, it may also be relevant that it was significantly more difficult to recruit patients for the “after” group. It is possible that milder cases of responsive behavior with knowledge of STI training in this group were already “resolved” on the ward without being reported to the study nurses thus resulting in the differences in the two groups. The incidental higher BPSD burden in the after-implementation group, represented by higher delirium rate and increased cognitive impairment, may have masked the positive effects of STI on NPI-Q-12 to some extent. However, in a linear mixed model analysis, an effect of delirium could also be detected but this effect seems to be very small.

In addition, even in the before-implementation group, there was already a substantial NPI-Q-12 improvement in 69% of the patients which can be interpreted as an indication that also in the before-group the interventions used in dealing with BPSD were already quite effective.

This result is in marked contrast to previous studies with STI which, unlike the present study, were conducted exclusively in nursing homes. A double-blinded randomized interventional study in nursing homes found significantly less discomfort in the STI group and more frequently behavioral symptoms returned to baseline (17). A cluster randomized controlled trial with 288 residents suffering from advanced dementia also showed an improvement in their behavior under strict application of the STI (39). Numerous publications in recent years favor such structured investigations of causes including non-pharmacological and pharmacological treatment strategies (40, 41).

### Non-pharmacological Treatment

In general, non-pharmacological interventions are considered as a first-line treatment option whenever possible with effect sizes that are similar to pharmacological approaches but with a lower risk of adverse events and often simpler application (40–42). However, the quality of evidence for such non-pharmacological interventions must be assessed as low (43).

In the study presented here, significantly more mobilizations and changes of position were performed for the patients in the after-implementation group. Likewise, nurses offered drinks and food more frequently in the context of responsive behavior. These changes can be seen as a possible success of the intervention. Successful increases of non-pharmacological interventions have also been described by other authors. Kovach et al. saw a clear increase in non-pharmacological treatments from pre- to post-testing through the use of STI (44). The most frequently used non-pharmacological comfort intervention at both pre- and post-testing times were soothing verbal communication and touch, movement, and sensory stimulation, respectively. However, this was not confirmed in a subsequent study (17).

### Pharmacological Treatment

We evaluated medications most commonly used in responsive behavior such as antipsychotics, antidepressants, sedatives, and anxiolytics (45).

No increase in analgesic use was demonstrated. The reason for the absence of a significant increase in analgesics could have been the low level of pain (median = 3) in both study groups. Given this low pain intensity, analgesic administration may not be as critical as originally expected. Our hypothesis was, among other things, that pain is one of the most important triggers for BPSD and an increase in analgesic consumption had been predicted when recognizing the problem. However, this could not be demonstrated which was possibly due to the relatively low pain intensity. The higher number of mobilizations, positioning, offering food or drink according to STI could be more useful and effective interventions. However, it should be noted that this statement is made against the background of existing adequate pain therapy. A non-structured pain assessment might have yielded different results. In both groups, almost 50% of the patients had already initially received a WHO step III analgesic and the proportion of patients treated with analgesics was over 80% thus possibly representing a special feature of hospital setting compared to nursing home environments. For example, Pieper et al. saw a significantly lower proportion of pain patients (50%) in nursing homes whereby the initial use of opiates was only 8% (46).

In contrast, Kovach et al. found significantly more use of analgesics in STI intervention compared to the control group (17) thus confirming an older study (44). A systematic clarification of the cause of BPSD improved pain management associated with responsive behavior more effectively than a non-stepwise approach (46). A recent consensus statement clearly prioritizes treatment with an analgesic over treatment with an antipsychotic (40).

Use of antipsychotics was even higher in the after-implementation group when considering the changes over the whole 4-day observation period in our study. One might assume that this increase is related to the random higher incidence of delirium and cognitive impairment in the after-implementation group. A linear mixed model analysis then also showed an influence of delirium on NPI-Q-12 values but its plain effect was ultimately negligible. However, this fact must be taken into account when interpreting the results. No correlation was found for cognitive impairment. In contrast, Pieper et al. found that gradual interventions in nursing home residents were successful without increasing psychotropic drug use (47).

### Feasibility

The study was able to show that introduction and application of an adapted version of STI is feasible in a hospital setting too. Almost 96% of the nursing staff in the selected sample wards were trained in the use of STI and its application. STI adaptations, especially regarding pain and its assessment but also regarding possible interventions in the hospital environment, were successfully implemented in the clinical routine. As part of the study, a curriculum and a pocket guide with the most important information regarding STI were developed and handed out to each nurse. In addition, a supporting e-version of STI with intervention suggestions was developed for use in everyday clinical practice.

## Strengths and Weaknesses

To the best of our knowledge, this is the first time that STI, originally developed for nursing home residents, has been applied and evaluated in an acute hospital setting (3). The transfer of STI into a new “acute hospital” setting and the implementation in a hospital routine characterized by time pressure and a high workload, where nurses are particularly affected by PWD with BPSD in their daily work (3) are certainly among the strengths of the study.

However, our study has some limitations which should not be left unmentioned. Due to the introduction of a new approach to BPSD with a before-after comparison, we decided against conducting a randomized controlled trial for several reasons. As the study was planned monocentrically, mixing effects, such as an exchange of relevant study information between the groups, could not be excluded with sufficient certainty. Furthermore, blinding for interventions was not possible. RCTs usually stand for a strictly defined equality of treatment which was not the case in the present study. Instead, the therapy was oriented to meet the individual needs of those affected and was formed from a plethora of possible interventions. Furthermore, it was never planned to conduct a confirmatory study as this would only be achievable with an RCT. On the other hand, the lack of randomization could bias the results in an unrepresentative direction. Possible confounders may have been overlooked and could explain the differences found between the groups. In order to provide an estimate of this effect in terms of delirium and cognition, we performed a linear mixed model analysis. However, we also know from Sessler and Imrey (48) that an inter-period comparative design, as in the current study, uses temporally proximal controls which are highly similar to those exposed to the intervention which may somewhat mitigate this effect.

In addition, the following limitations can be discussed: although the proportion of trained nurses was very high (95.8%), it is conceivable that nurses who were not trained were also used in each of the four STI application days. However, given the high training rate, we believe that any resulting effects from lack of training are negligible. It is also conceivable that the observation period of 4 days, based purely on clinical experience, may not have been long enough to observe any significant differences between the two observation groups regarding BPSD. The measured pain scores of the participants were possibly too low to expect significant changes regarding analgesics. There were no specifications such as a minimum pain score as an entry criterion. To sum up, the present study only considered patient-related factors such as pain, cognition, and physical function. BPSD can, however, be influenced by many other factors such as a patient's environment (e.g., living situation), socioeconomic factors (e.g., school education) and by reactions to the interaction with the caregiver (e.g., caregiver burden), which were not considered in the current study (10, 49).

## Conclusion

Even though the study ultimately failed to show a significant reduction in BPSD, the introduction of stepwise clarification of possible causes of BPSD, especially pain, demonstrates an attractive approach to BPSD management. STI seems to at least raise awareness of underlying causes of BPSD and increase the use of non-pharmacological interventions. In this respect, STI seems applicable and beneficial in the treatment of BPSD, also for hospitalized patients. Nevertheless, further studies need to follow to conclusively clarify the value of STI in an acute hospital setting.

## Data Availability Statement

The raw data supporting the conclusions of this article will be made available by the authors, without undue reservation.

## Ethics Statement

The studies involving human participants were reviewed and approved by Ethics Committee, University Bonn, Germany (No. 252/15). The patients/participants provided their written informed consent to participate in this study.

## Author Contributions

AL initiated and conceptualized the study idea, formulated the hypothesis, carried out the study planning, conducted the training, supervised the data collection, analyzed the data, and drafted and revised the manuscript. MB was involved conceptualized the study idea, formulated the hypothesis, carried out the assessment and collected data, analyzed the data, and drafted and revised the manuscript. BM was extensively involved in the statistical planning and analyses and also drafted and revised the manuscript. LR initiated and conceptualized the study idea, was involved in the study planning, and drafted and revised the manuscript. IG initiated and conceptualized the study idea, supported in the context of pain issues, designed the curriculum, conducted the training, and drafted and revised the manuscript. All authors participated in critical revision of the article for intellectual content, meet the criteria for authorship stated in the Uniform Requirements for Manuscripts Submitted to Biomedical Journals, and have read and approved the final manuscript.

## Funding

The study was funded by the Robert Bosch Foundation within the framework Dementia in acute hospitals (Grant number: 32.4.1365.0103.0). The sponsor had no role in the design, methods, subject recruitment, data collection, analysis or preparation of the manuscript.

## Conflict of Interest

The authors declare that the research was conducted in the absence of any commercial or financial relationships that could be construed as a potential conflict of interest.

## Publisher's Note

All claims expressed in this article are solely those of the authors and do not necessarily represent those of their affiliated organizations, or those of the publisher, the editors and the reviewers. Any product that may be evaluated in this article, or claim that may be made by its manufacturer, is not guaranteed or endorsed by the publisher.
